# The Use of Automated Quantitative Analysis to Evaluate Epithelial-to-Mesenchymal Transition Associated Proteins in Clear Cell Renal Cell Carcinoma

**DOI:** 10.1371/journal.pone.0031557

**Published:** 2012-02-21

**Authors:** Fiach C. O'Mahony, Dana Faratian, James Varley, Jyoti Nanda, Marianna Theodoulou, Antony C. P. Riddick, David J. Harrison, Grant D. Stewart

**Affiliations:** Edinburgh Urological Cancer Group, Division of Pathology, Institute of Genetics and Molecular Medicine, University of Edinburgh, Edinburgh, United Kingdom; National Taiwan University Hospital, Taiwan

## Abstract

**Background:**

Epithelial-to-mesenchymal transition (EMT) has recently been implicated in the initiation and progression of renal cell carcinoma (RCC). Some mRNA gene expression studies have suggested a link between the EMT phenotype and poorer clinical outcome from RCC. This study evaluated expression of EMT-associated proteins in RCC using *in situ* automated quantitative analysis immunofluorescence (AQUA) and compared expression levels with clinical outcome.

**Methods/Principal Findings:**

Unsupervised hierarchical cluster analysis of pre-existing RCC gene expression array data (GSE16449) from 36 patients revealed the presence of an EMT transcriptional signature in RCC [E-cadherin high/SLUG low/SNAIL low]. As automated immunofluorescence technology is dependent on accurate definition of the tumour cells in which measurements take place is critical, extensive optimisation was carried out resulting in a novel pan-cadherin based tumour mask that distinguishes renal cancer cells from stromal components. 61 patients with ccRCC and clinical follow-up were subsequently assessed for expression of EMT-associated proteins (WT1, SNAIL, SLUG, E-cadherin and phospho-β-catenin) on tissue microarrays. Using Kaplan-Meier analysis both SLUG (p = 0.029) and SNAIL (p = 0.024) (log rank Mantel-Cox) were significantly associated with prolonged progression free survival (PFS). Using Cox regression univariate and multivariate analysis none of the biomarkers were significantly correlated with outcome. 14 of the 61 patients expressed the gene expression analysis predicted EMT-protein signature [E-cadherin high/SLUG low/SNAIL low], which was not found to be associated to PFS when measured at the protein level. A combination of high expression of SNAIL and low stage was able to stratify patients with greater significance (p = 0.001) then either variable alone (high SNAIL p = 0.024, low stage p = 0.029).

**Conclusions:**

AQUA has been shown to have the potential to identify EMT related protein targets in RCC allowing for stratification of patients into high and low risk groups, as well the ability to assess the association of reputed EMT signatures to progression of the disease.

## Introduction

Renal cell carcinoma (RCC) is the most deadly all the urological malignancies [1]. In the UK 8,228 cases were diagnosed in 2007 and there were 58,240 new cases in the USA in 2008 [2]. The reported incidence of RCC is increasing at a rate of 2.5% though partially because of serendipitous identification due to improved imaging techniques [3]. Localised and locally advanced tumours are typically treated with nephrectomy; however, in 20–40% of cases the disease will recur [4] and currently there is no curative treatment for metastatic RCC. RCC is not a single disease entity but a heterogeneous collection of cancers which arise in the kidney, each driven by different genes and pathways [5], [6]. In addition, each cancer shows different clinical and pathological features, with distinct patterns of origin within the nephron, local invasion, and distant metastases [7]. Such complexity is thought to be one of the reasons accounting for the low level of success in the treatment of metastatic RCC [8].

The most common histological subtype of RCC is clear cell RCC (ccRCC) which accounts for 70–80% of sporadic cases [9]. Loss-of-function mutations of the von Hippel Lindau (VHL) tumour suppressor gene occur in at least two-thirds of sporadic ccRCC cases [10], as well as accounting for hereditary ccRCC. Even within ccRCC, subgroups based on gene expression clusters associated with differential prognostic outcomes, have been identified [11], [12], [13] allowing for the potential to study ccRCC in greater detail.

In common with some other cancers epithelial-to-mesenchymal transition (EMT) has been identified as potentially playing a significant role in RCC [11], [14], [15]. EMT which is well characterised in embryogenesis and wound healing, and has also been linked to pathogenesis and tumour invasion [16]. Tun *et al* proposed a model for RCC pathogenesis in which the development biological process, mesenchymal-to-epithelial transition (MET), is reversed resulting in EMT and dedifferentiation [15]. MET is the process by which the kidney, which is mesenchymal in origin, develops. In addition, Brannon *et al* used consensus clustering of gene expression data to show that ccRCC could be divided into two subtypes with opposing EMT phenotypes, with prognosis being poorer for the EMT phenotype [11]. The work by Brannon and colleagues supported a previous study which found that the activation of a wound healing gene expression signature in ccRCC was associated with the features of EMT and a poorer survival in ccRCC patients [14].

The identification of genes of interest related to EMT in ccRCC pathogenesis allows for the translation of such genetic targets into proteins of interest for use in the development of clinically relevant tests. Despite the wide range of protein biomarkers available for the study of ccRCC [8], [17] there is a lack of published data on the use of protein expression in relation to EMT in ccRCC. Immunohistochemistry (IHC) is a key molecular pathology technique used in diagnosis, prognosis and prediction of treatment outcomes in RCC in the clinic. However, classical IHC itself is only semi-quantitative at best. The recent development of automated quantitative analysis (AQUA) of immunofluorescence has allowed accurate and sensitive *in situ* protein quantification of protein levels [18]. Despite the increasing use of AQUA for the study of prognostic and predictive biomarkers in other cancers including breast, ovary, and melanoma [19], [20], its use in the study of ccRCC is scarce [21], [22], most likely because many renal cancer cells are negative for cytokeratins which are used in the other epithelial cancers to precisely distinguish tumour cells from stroma.

In this study quantitative immunofluorescence was utilised for the first time to test the hypothesis that EMT related protein markers and a reputed EMT signature, selected following analysis of published gene expression data and review of the literature, are of prognostic significance in ccRCC in a population of ccRCC patients.

## Materials and Methods

### Gene expression analysis of GE16449 dataset

DNA microarrays dataset accession number GS16449 [11], which contained tumour samples from 36 patients with ccRCC was downloaded from the Gene Expression Omnibus public database repository. This dataset which is MIAME compliant was generated using Agilent Whole Human Genome (4×44 k) Oligo Microarrays. Data was normalized using the robust multichip average (RMA) technique. The probesets for EMT gene targets used in this analysis are listed in Table S1. Transcript levels were mean-centred and analyzed using unsupervised cluster analysis (average linkage clustering, Spearman's rank correlation distance metric) to establish relationships between the transcripts.

### Study population and design

The study population consisted of 61 formalin-fixed, paraffin-embedded tumour samples from patients with ccRCC who underwent either partial or radical nephrectomy for suspected RCC at the Department of Urology, Western General Hospital, Edinburgh between 2001 and 2003. The study was approved by the Lothian Research Ethics Committee (08/S1101/41). No informed consent (written or verbal) was obtained for use of retrospective tissue samples from the patients within this study, most of whom were deceased, since this was not deemed necessary by the Ethics Committee, who waived the need for consent. All samples were anonymised. Table 1 summarises the characteristics of the study population. Progression free survival (PFS) was calculated from the date of nephrectomy to the date of progression of the RCC (diagnosed on cross-sectional imaging), or to the date of last follow-up (censored).

**Table 1:** Clinicopathological characteristics of ccRCC patients included in study TMA (n = 61).

Characteristic		Number/Variable	%
**Age**	*Median (years)*	65.4	N/A
	*Range (years)*	28.1–86.3	N/A
**Sex**	*Female*	23	37.7
	*Male*	38	62.3
**Grade**	*1*	1	1.6
	*2*	33	54.1
	*3*	19	31.1
	*4*	8	13.1
**Nodal status at surgery**	*Nx*	48	78.7
	*N0*	10	16.4
	*N1*	1	1.6
	*N2*	2	3.3
**Metastatic status at surgery**	*M0*	49	80.3
	*M1*	12	19.7
**T Stage**	*1*	24	39.3
	*2*	12	16.4
	*3*	23	41.0
	*4*	2	3.3
**Tumour Size (cm)**	*Median*	6.8	N/A
	*Range*	3.0–16.5	N/A
**RCC PFS (months)**	*Median*	8.9	N/A
	*Range*	0.0–91.5	N/A

### Tissue microarray (TMA) construction

In brief, following H&E staining of representative tumour blocks from all 61 patients, three replicate TMAs with core diameters of 0.6 mm were constructed using established techniques [23]. A separate tissue microarray (TMA) was created from 18 ccRCC samples for the purposes of tumour mask optimisation (Table S2).

### Tumour Mask Optimisation

For each core, areas of tumour are differentiated from stroma by creating an epithelial tumour mask. As the collection of target antibodies chosen for study included those from mouse and from rabbit, tumour masks complementary to both sets were needed. The following antibodies were tested as part of the tumour mask optimisations: pan-cadherin *(Cell Signalling 4068, rabbit*), cytokeratin (CK; *Dako Z0622, rabbit*), HRP-streptavidin (*Invitrogen 43-4323*), biotin (*Dako E0464, rabbit*), CK5/6/8/18 (*Novocastra NCL-CK5/6/8/18, mouse*), CD10 (*Novocastra NCL-CD10-270, mouse*), RCC (*Novocastra NCL-RCC, mouse*), EMA (*Dako M0613, mouse*), CK (*Dako M3515, mouse*), vimentin (*Sigma V6630, mouse*), pan-cadherin (*Sigma-Aldrich C1821, mouse*) and IL-15 (Abcam *ab55282, mouse*). Table S3 details antibody dilutions and antigen retrieval solutions employed. Masking single antibodies and antibody combinations were assessed based on their ability to: (i) distinguish between tumour and stromal areas, (ii) bind homogenously across the entire core, and (iii) bind both cell membrane and cytoplasm.

### Immunofluorescence

Immunofluorescence was performed using methods previously described [24]. Briefly, 3 µm TMA slides were deparaffinized and antigen retrieved by pressure cooking in either Sodium Citrate buffer pH 6.0 or Tris-EDTA (TE) buffer pH 9.0 for 5 minutes, following which the TMA sections were blocked using 3% H_2_O_2_ and serum-free protein block. The slides were then incubated for 1 hour at room temperature with the one of the following monoclonal antibodies E-cadherin (BD Sciences, 610181) SLUG (LifespanBio, LS-C30318), SNAIL (Abcam, ab17732), WT1 (Genetex, GTX15249) and phospho-β-catenin (Cell Signalling, 9561). After treatment with the target antibodies, TMA sections were incubated overnight at 4°C with pan-cadherin (Cell Signalling, 4068, 1∶100) or a combination of pan-cadherin (Sigma-Aldrich, C1821, 1∶750) and CK5/6/8/18 (Novocastro, 6003168, 1∶100) to mask tumour areas for rabbit and mouse based target antibodies respectively (see results section for details). TMA sections were subsequently incubated for 1.5 hours with secondary antibodies: Alexa Fluor 555–conjugated antibody (tumour mask) and horseradish peroxidase–decorated dextran polymer backbone (EnVision, Dako) to amplify the target protein. DAPI (4′, 6-diamidino-2-phenylindole) counterstain was used to visualise the nuclei and Cy-5-tyramide used to detect target to allow compartmentalised analysis of tissue sections. A summary of the experimental conditions can be seen in Table 2.

**Table 2:** The primary antibodies used during the study and the experimental conditions.

Target/Dilution	Supplier	Species	Antigen Retrieval Solution	Tumour Mask (Antibody/Dilution)
**E-cadherin (1 in 1500)**	BD Sciences	Mouse	Sodium Citrate pH 6.0	Pan-cadherin (1 in 100)
**SNAIL (1 in 800)**	Abcam	Rabbit	TE Buffer pH 9.0	Pan-cadherin and CK5/6/8/18 (1 in 750 and 1 in 100)
**SLUG (1 in 1000)**	Lifespan Bio	Rabbit	TE Buffer pH 9.0	
**WT1 (1 in 100)**	Genetex	Rabbit	TE Buffer pH 9.0	
**phospho-β-catenin (1 in 25)**	Cell Signalling	Rabbit	TE Buffer pH 9.0	

### AQUA automated image analysis

A detailed description of the AQUA methodology is available elsewhere [24]. In brief, monochromatic images of each TMA core were captured at 20× objective using an Olympus AX-51 epifluorescence microscope, and high-resolution digital images were analyzed by the AQUAnalysis software. A binary epithelial mask was created from the optimised tumour mask antibodies to create an image of each TMA core. If the epithelium comprised <5% of total core area, the core was excluded from analysis. Similar binary masks were created for cytoplasmic and nuclear compartments based on DAPI staining of nuclei. Target expression was quantified by calculating the Cy5 fluorescent signal intensity on a scale of 0 to 255 within each image pixel, and the AQUA score was generated by dividing the sum of Cy5 signal within the epithelial mask by the area of the cytoplasmic compartment.

### Statistical analysis methods

AQUA scores were initially averaged from replicate cores, and mean centred for each array replicate. To reduce type I error that can result from using the minimum *p* value method for determining the cutpoint value for target expression in Kaplan-Meier analysis [25] X-Tile was used; this allows determination of an optimal cutpoint while correcting for the use of minimum *p* statistics [26]. Overall survival was subsequently assessed by Kaplan-Meier analysis with log rank for determining statistical significance. As the continuous quantitative values were not normally distributed non-parametric statistical analysis were conducted. Multiple continuous, non-parametric variables were compared with the Kruskal-Wallis test. To correct for the error (Type I and II) due to multiple comparisons, significant P value thresholds were corrected by using the Bonferroni Correction. Univariate and multivariate analysis of biomarkers and clinicopathological variables was calculated using Cox regression analysis. c indexes were calculated using ROC-AUC. All calculations and analyses were two tailed where appropriate were conducted using SPSS 14.0 for Windows (SPSS, Inc., Chicago, IL, USA).

## Results

### Cluster analysis of ccRCC gene expression indicates the existence of EMT and non-EMT phenotypes

**Figure 1:**
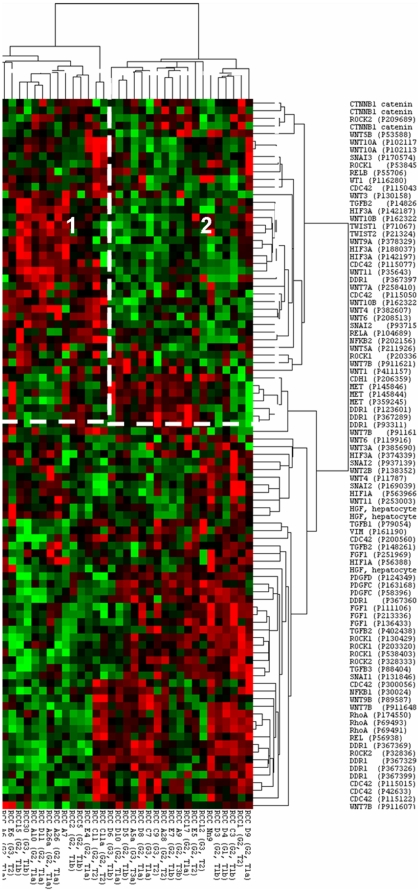
Unsupervised hierarchical clustering of the GSE16449 microarray dataset, using the probe-sets of interest (Table S1). Cluster 1 represents the group expressing the EMT signature and increased Wnt signalling, whereas the Cluster 2 illustrates the non-EMT cluster group. (green = low expression, red = high expression).

Unsupervised hierarchical cluster analysis of the DNA microarray set accession number GS16449 [11] using the EMT relevant probe sets listed in Table S1 identified the presence of two main clusters: an “EMT cluster” (Cluster 1, Figure 1) with low expression of CDH1 (E-cadherin), high expression of SNAI3 (SNAIL homolog 3) and high expression of SNAI2 (SNAIL homolog 2, SLUG); and “non-EMT cluster” with reciprocal expression of these genes (Cluster 2, Figure 1). Increased Wnt signalling indicated by high CTNNB1 [β-catenin] expression and high expression of WT1 were also observed in the EMT cluster. As a result E-cadherin, SNAIL, SLUG, WT1 and phospho-β-catenin proteins were selected for study at a protein level.

### Tumour Mask Antibody Optimisation

**Figure 2:**
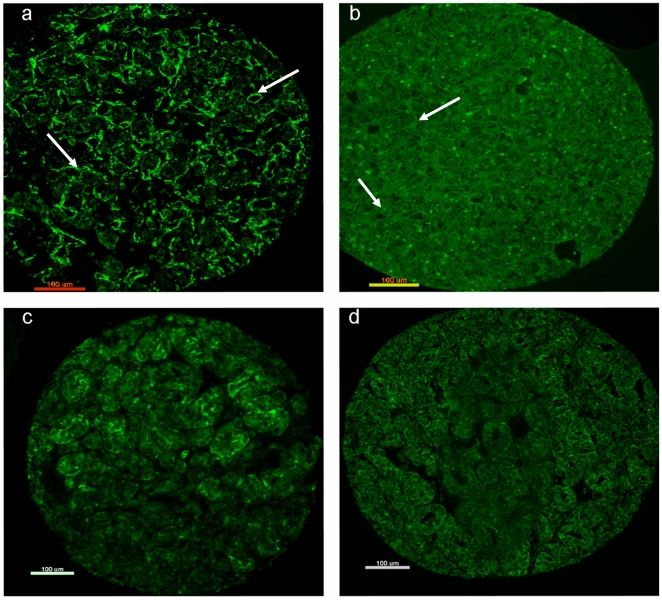
Images of selected tumour masking options. (a) Vimentin (stroma stained) (b) Renal cell marker (stroma and epithelia stained) (c) Pan-cadherin and CK5/6/8/18 (representative tumour mask for optimised mouse tumour mask) (d) Pan-cadherin (representative tumour mask for rabbit tumour mask). White arrows indicate areas of non specific binding.

**Figure 3:**
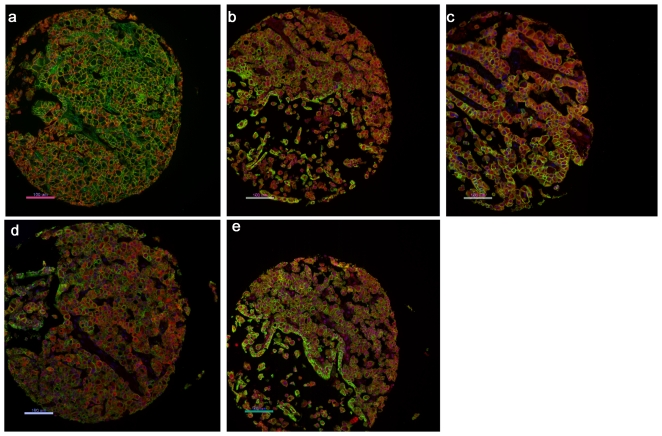
AQUA quantitative image analysis. DAPI counterstain (blue) was used to identify nuclei, pan-cadherin antibody (rabbit) or a combination of pan-cadherin and pan-cytokeratin antibodies (mouse) to identify infiltrating tumour cells (green) and Cy-5-tyramide for detection of following target proteins (red) (a. E-cadherin, b. SNAIL, c. SLUG, d.WT1 and e. phospho-β-catenin).

In order to study the differential protein expressions of targets identified from the microarray data it was necessary to establish two tumour masking protocols, to complement the species in which the target antibodies were raised. 738 AQUA images of 41 different tumour mask combinations used to treat the optimisation TMA (including 18 ccRCC tissue samples) were analysed (Figure 2 illustrates examples). Of the antibodies tested a 1 in 100 dilution of pan-cadherin (rabbit) (Figure 2d) best identified the tumour area compared to other rabbit raised antibodies, binding strongly to epithelial tumour areas with minimal stromal binding. This was in contrast to the mouse antibody vimentin which only bound stroma (Figure 2a) or renal cell marker (RCC) which was non-selective, binding both tumour and stromal areas equally (Figure 2b). The best of the mouse derived antibodies was the pan-cadherin and CK5/6/8/18 combination which allowed for selective binding to the epithelial tumour area with minimal stromal binding (Figure 2c). These optimised antibodies were subsequently used to detected and quantify the markers of interest on the TMA (Figure 3a–e).

### Automated quantitative analysis of individual targets and their association with clinicopathological parameters

Associations between the mean AQUA scores (Au) of the individual protein targets and the following clinicopathological variables were performed: grade, stage, histology, metastatic and lymph node status (Kruskal–Wallis test; Table S4). Before correction the only significant association was between E-cadherin and tumour grade (p = 0.032), however when corrected for multiple comparisons only Grade 3 versus 4 was found to be significant (p = 0.004) at the recalculated significance level (p≤0.0083). Using Spearman rank correlation coefficient, no significant correlations were detected when clinicopathological variables were tested against the EMT biomarkers (Table 3). SLUG SNAIL and E-Cadherin all gave c-indexes greater then 0.5 when tested for PFS indicating that the X-tile defined cut off points were at least better then chance at patient stratification (Table S5).

**Table 3:** Spearman's Correlation Test of clinicopathological variables with EMT biomarkers.

	Variables		Slug Nuclear	SNAIL Nuclear	E-Cadherin Cytoplasm
**Spearman's rho**	**Node**	*Correlation Coefficient*	0.043	−0.043	0.022
		*Sig. (2-tailed)*	0.742	0.742	0.869
	**Grade coded**	*Correlation Coefficient*	0.049	−0.191	−0.169
		*Sig. (2-tailed)*	0.709	0.140	0.193
	**T stage**	*Correlation Coefficient*	−0.199	−0.130	−0.009
		*Sig. (2-tailed)*	0.125	0.317	0.943
	**Metastases**	*Correlation Coefficient*	−0.194	−0.194	0.018
		*Sig. (2-tailed)*	0.133	0.133	0.893

n = 61 for all analysis.

### Association between clinicopathological variables and survival

**Figure 4:**
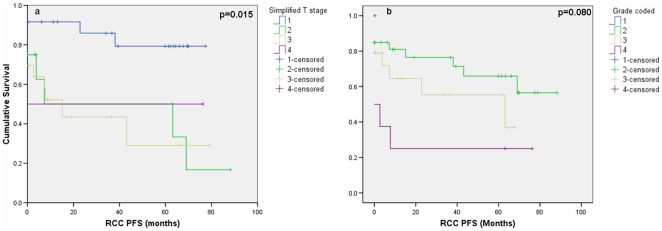
Progression free survival (PFS) curves. (a) stage and (b) grade using Kaplan-Meier methods. Note that 13 patients were classed as having 0 months PFS due to having metastatic disease at the time of nephrectomy.

The effect of tumour stage and grade on PFS were assessed (Figure 4). In the current study only stage was significantly associated with PFS (p-value = 0.015); increasing stage resulting in decreasing PFS. When the significance association of stage with PFS was corrected for multiple comparisons (p≤0.008), only T1 versus T2 (p = 0.008) and T1 versus T3 (p = 0.002) were significant. At the univariate level stage, grade and presence of metastases were all significant indicators of progression free survival (Table 4). Despite this only metastases remained significant after multivariate analysis (Table 5).

**Table 4:** Univariate Cox Regression analysis of clinicopathological variables and EMT biomarkers against PFS.

Variables	p value	Hazard Ratio	95% CI for Hazard Ratio	
			*Lower*	*Upper*
**Grade**	0.020*	1.818	1.100	3.004
**Stage**	0.012*	1.690	1.212	2.547
**Node**	0.102	3.515	0.779	15.859
**Metastases**	0.0002**	47.577	6.344	356.788
**E-Cadherin Cytoplasm**	0.417	1.144	0.827	1.583
**SNAIL Nuclear**	0.081	0.511	0.240	1.086
**Slug Nuclear**	0.194	0.570	0.244	1.332

*Significant at p<0.05 level.

**Significant at p<0.001 level.

**Table 5:** Multivariate Cox Regression Analysis of clinicopathological variables and EMT biomarkers against PFS.

Variables	p value	Hazard Ratio	95% CI for Hazard Ratio	
			*Lower*	*Upper*
Grade	0.762	1.293	0.245	6.810
Stage	0.605	1.184	0.645	2.249
Node	0.407	1.240	0.746	2.062
Metastases	0.001*	33.114	3.949	277.665
E-Cadherin Cytoplasm	0.672	1.080	0.756	1.544
SNAIL Nuclear	0.657	0.809	0.316	2.067
Slug Nuclear	0.902	0.934	0.317	2.753

*Significant at p<0.05 level.

### Independent validation of EMT signature in ccRCC at the protein level using AQUA

**Figure 5:**
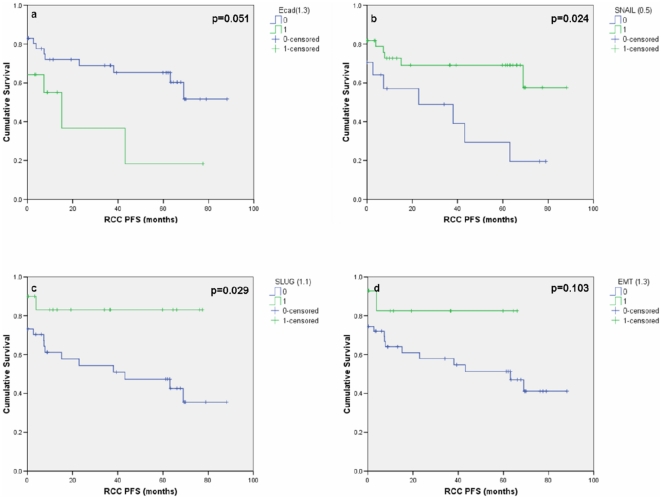
Survival curves based on AQUA protein expression cut-off points. (a) E-cadherin expression, (b) SNAIL expression, (c) SLUG expression and (d) EMT phenotype versus progression free survival (PFS). Note that 13 patients were classed as having 0 months PFS due to metastatic disease at the time of nephrectomy.

A cut-off point of the AQUA score that best predicted short and long PFS for each of the individual targets was defined using the minimum p-value method [26]. Generation of these cut-off points enabled the association between protein expression and clinical outcome to be established. As a result cut-off points for E-cadherin, SNAIL and SLUG were used to divide AQUA scores into either being low or high protein expression. Cut-off points for WT1, phospho-β-catenin were not able to significantly divide AQUA scores. These cut-off points were then used to create Kaplan-Meier survival curves (Figure 5). SLUG (Figure 5c) and SNAIL (Figure 5b) were found to be significant markers of PFS (p = 0.029 and p = 0.024 (log rank Mantel-Cox test) respectively) with high expression of these proteins being associated with improved survival. In contrast E-cadherin (Figure 5a) did not reach significance (p = 0.051) and high expression was associated with a poorer prognosis. The EMT biomarkers didn't reach significance using Cox regression after either univariate (Table 4) or multivariate analysis (Table 5).

An EMT-protein signature defined as [E-cadherin high/SLUG low/SNAIL low] derived from the gene expression data was assessed for its ability to stratify patients into low and high risk groups. Using this criterion 14 of the 61 ccRCC patient samples were identified as showing the EMT phenotype. There was no significant association between presence of the EMT-protein signature and PFS (p = 0.103, log-rank test; Figure 5d).

**Figure 6:**
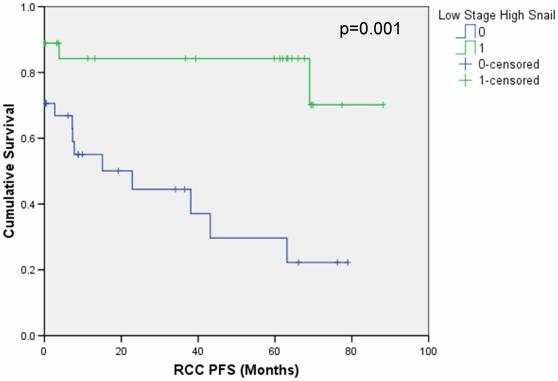
Progression free survival (PFS) curves based on combination of low stage and high SNAIL protein expression. Note that 13 patients were classed as having 0 months PFS due to metastatic disease at the time of nephrectomy.

In an effort to improve the significance of individual biomarkers and clinicopathological variables combinations of the independently significant biomarkers, i.e. high expression of SLUG or SNAIL, in addition to grade and stage dichotomised to either being low (grade 1 or 2, stage 1 or 2) or high (grade 3 or 4, stage 3 or 4) were tested and analysed using the Kaplan Meier method (Table 6) [27], [28]. Of the combinations tested only low grade + low stage (p = 0.025) and low stage + high SNAIL (p = 0.001) (Figure 6) produced significant p values. Both of these combinations gave more significant p values then the individual parameters alone, with the combinations associated with improved progression free survival.

**Table 6:** Combinations of the clinicopathological variable stage and grade (dichotomised into high and low) and high expression of SLUG and SNAIL.

SLUG	SNAIL	Stage	Grade	Number of Patients Meeting Criteria. Total = 61	Log Rank (Mantel-Cox) p value
High				20	0.029*
	High			44	0.024*
		Low		25	0.029*
			Low	27	0.036*
High	High			18	0.082
High	High	Low	Low	8	0.121
		Low	Low	22	0.025*
High	High	Low		13	0.235
High	High		Low	10	0.236
High		Low		13	0.253
High			Low	20	0.380
	High		Low	28	0.075
	High	Low		27	0.0008**

*Significant at p<0.05 level.

**Significant at p<0.001 level.

## Discussion

The current study describes the expression of EMT related proteins in ccRCC patients using quantitative immunofluorescence AQUA technology. This study demonstrated detailed optimisation of AQUA for use in RCC research. Although an EMT signature was identified at the gene expression level, which was in agreement with other published ccRCC EMT related gene expression studies [11], [14], no significant association was found between presence of the EMT-protein signature and worse prognosis in ccRCC patients. A combination of low stage and high SLUG expression was associated with a good clinical outcome of greater significance than either stage or SLUG expression alone.

In the study we have defined a protocol for distinguishing epithelial tumour cells in RCC from stroma. Unlike the majority of previous AQUA studies in a number of different tumour types, which incorporate cytokeratin in the tumour mask [22], the current study used pan-cadherin antibodies as many RCC are cytokeratin negative [29]. This ability to diversify from cytokeratin antibodies in addition to being able to tailor the tumour mask to the species in which the target antibodies have been raised highlights the flexibility of AQUA as a system for studying protein expression. A previous RCC study demonstrating AQUA used a non-cytokeratin based tumour mask, specifically a vimentin/EMA/CD10 combination [21]. In our experience the vimentin/EMA/CD10 combination bound to both the stroma and epithelial tumour area, unlike the optimal tumour masking strategies developed following our optimisation strategy.

Recent RCC gene expression studies have implicated EMT in RCC pathogenesis [11], [14], [15]. An inherent drawback of any gene expression profiling technique is that, despite the high number of potential targets available for study, steady-state levels of RNA are not necessarily reflective of the final steady-state level of the functional protein translation product of the mRNA [30]. In contrast protein expression determinations by methods such as AQUA give an accurate insight into the relative levels of the selected proteins in any given study. Despite this no dedicated protein expression studies of EMT in RCC exist. In the current study, AQUA analysis was successful in the accurate detection of EMT related proteins including E-cadherin, SLUG, SNAIL, WT1 and phospho-β-catenin in ccRCC. Of these proteins SLUG and SNAIL were able to categorise patients into high and low risk for PFS to a significant degree (p<0.05), warranting further study into their potential use as putative prognostic protein markers in ccRCC. The lack of the expected statistically significant association of patient survival with E-cadherin, WT1, and phosho-β-catenin may be due to relatively small number of patients samples included in the study, but in any event suggest that these measurements would be of limited value in a clinical setting for individual patients.

Of particular interest was the significant association of high expression of SLUG and SNAIL with improved survival and the association of high E-cadherin expression with worse survival in contrast to published data in RCC [11], [14] and other cancers [31], [32]. Despite this contradiction a recent similar RCC study investigating the association of SLUG, SNAIL and E-cadherin in addition to other markers with improved survival, showed that high SLUG was significantly associated with improved survival, in contrast to SNAIL which was negative associated with survival while E-cadherin association wasn't significant [33]. Of the 61 patient samples in the current studies, 14 patients had the combined EMT-protein signature of low E-cadherin and high expression of the E-cadherin repressors SLUG and SNAIL. In contrast to other studies of EMT in ccRCC, in the current study the EMT-protein signature was not associated with a worse prognosis [11], [14], [15]., although this event has been identified in a recent breast cancer study [34].

This study demonstrates the application and optimisation for RCC of AQUA, a medium throughput method of *in situ* quantification of protein using FFPE tissue. Furthermore, it has been demonstrated that the transcription factors SNAIL and SLUG are potential prognostic protein markers in ccRCC, especially in combination with clinical parameters i.e. tumour stage. The association of an EMT phenotype with poor clinical outcome, previously demonstrated at the transcript level, has not been confirmed by this protein study. A larger ccRCC sample set along the lines described by Pepe *et al* [35] and the expansion of the targets to include further EMT related proteins would be necessary to further elucidate the role of EMT in ccRCC.

## Supporting Information

Table S1 **Probeset for EMT targets.**
(DOC)

Table S2 **Clinicopathological characteristics of ccRCC patients included in the optimisation TMA (n = 18).**
(DOC)

Table S3 **The antibodies used during the tumour mask optimisation process and associated experimental conditions.** Chosen, optimised protocols highlighted in bold.
(DOC)

Table S4 **Comparison of patient pathology details and target Au scores with calculated uncorrected p-values (Kruskal–Wallis test, significance at p<0.05 level).** Only grade 3 versus grade 4 remained significantly associated with E-Cadherin after correction for multiple comparisons (p = 0.004, significance at P<0.0083 level).
(DOC)

Table S5 **Area under the receiver operating characteristic curve (AUC) c-indexes for each clinicopathological variable and EMT biomarker.**
(DOC)
