# Supplementary material for: The Use of Automated Quantitative Analysis to Evaluate Epithelial-to-Mesenchymal Transition Associated Proteins in Clear Cell Renal Cell Carcinoma
Source: PLoS One. 2012 Feb 21;7(2):e31557. doi: 10.1371/journal.pone.0031557 (PMC3283650; doi:10.1371/journal.pone.0031557)
Supplement: Table S1 — Probeset for EMT targets. (DOC) [file pone.0031557.s001.doc]

| HIF3A (P374339) | WNT5B (P53588) | FGF1 (P136433) |
| --- | --- | --- |
| HIF3A (P188037) | WNT9B (P89587) | RhoA (P69491) |
| HIF3A (P142187) | WNT4 (P11787) | RhoA (P69493) |
| HIF3A (P142197) | MET (P359245) | RhoA (P174550) |
| HIF1A (P56388) | MET (P145844) | ROCK2 (P328333) |
| HIF1A (P563966) | MET (P145846) | ROCK2 (P32836) |
| WNT6 (P208513) | SNAI2 (P169039) | ROCK1 (P538403) |
| WNT11 (P253003) | SNAI3 (P170574) | ROCK1 (P538459) |
| WNT11 (P35643) | SNAI2 (P937139) | ROCK1 (P203320) |
| WNT7B (P911607) | SNAI2 (P937158) | ROCK1 (P203362) |
| WNT7B (P911612) | SNAI1 (P131846) | ROCK1 (P130429) |
| WNT7B (P911621) | CTNNB1 (P29495) | ROCK2 (P209689) |
| WNT7B (P911648) | CTNNB1 (P29499) | CDC42 (P42633) |
| WNT10A (P102113) | CTNNB1 (P608330) | CDC42 (P115015) |
| WNT10A (P102117) | HGF (P93780) | CDC42 (P115043) |
| WNT6 (P119916) | HGF (P93787) | CDC42 (P115050) |
| WNT3 (P130158) | HGF (P944788) | CDC42 (P115077) |
| WNT2B (P138352) | NFKB1 (P30024) | CDC42 (P115122) |
| WNT10B (P162322) | NFKB2 (P202156) | CDC42 (P200560) |
| WNT10B (P162322) | RELA (P104689) | CDC42 (P300056) |
| WNT5A (P211926) | RELB (P55706) | WT1 (P116280) |
| WNT7A (P258410) | REL (P56938) | DDR1 (P93311) |
| WNT9A (P378329) | CDH1 (P206359) | DDR1 (P123601) |
| WNT4 (P382607) | FGF1 (P213336) | DDR1 (P367289) |
| WNT3A (P385690) | FGF1 (P111106) | DDR1 (P367326) |
| WNT1 (P411157) | FGF1 (P251969) | DDR1 (P367329) |
| DDR1 (P367360) | DDR1 (P367399) | PDGFD (P124349) |
| DDR1 (P367369) | VIM (P161190) | PDGFC (P163168) |
| DDR1 (P367397) | PDGFC (P58396) | TGFB3 (P88404) |
| TGFB1 (P79054) | TWIST2 (P21324) | TWIST1 (P71067) |

Supplementary Table 1. Probeset for EMT targets
