# Supplementary material for: The Use of Automated Quantitative Analysis to Evaluate Epithelial-to-Mesenchymal Transition Associated Proteins in Clear Cell Renal Cell Carcinoma
Source: PLoS One. 2012 Feb 21;7(2):e31557. doi: 10.1371/journal.pone.0031557 (PMC3283650; doi:10.1371/journal.pone.0031557)
Supplement: Table S2 — Clinicopathological characteristics of ccRCC patients included in the optimisation TMA (n = 18). (DOC) [file pone.0031557.s002.doc]

| Characteristic | | Number/Variable | % |
| --- | --- | --- | --- |
| Age | Median (years) | 66 | N/A |
|  | Range (years) | 40-78 | N/A |
| Sex | Female | 9 | 50 |
|  | Male | 9 | 50 |
| Grade | 1 | 0 | 0 |
|  | 2 | 9 | 50 |
|  | 3 | 4 | 22.2 |
|  | 4 | 5 | 27.8 |
| T Stage | 1 | 9 | 50 |
|  | 2 | 4 | 22.2 |
|  | 3 | 4 | 22.2 |
|  | 4 | 1 | 5.6 |

Supplementary Table 2. Clinicopathological characteristics of ccRCC patients included in the optimisation TMA (n=18).
