# Supplementary material for: The Use of Automated Quantitative Analysis to Evaluate Epithelial-to-Mesenchymal Transition Associated Proteins in Clear Cell Renal Cell Carcinoma
Source: PLoS One. 2012 Feb 21;7(2):e31557. doi: 10.1371/journal.pone.0031557 (PMC3283650; doi:10.1371/journal.pone.0031557)
Supplement: Table S3 — The antibodies used during the tumour mask optimisation process and associated experimental conditions. Chosen, optimised protocols highlighted in bold. (DOC) [file pone.0031557.s003.doc]

| **Species** | **Antibody** | **Supplier** | **Dilution** | **Antigen Retrieval Solution** |
| --- | --- | --- | --- | --- |
| **Rabbit** | ***Pan-cadherin*** | ***Cell Signalling*** | 1 in 10 | TE Buffer pH 9.0 |
|  |  |  | 1 in 20 |  |
|  |  |  | 1 in 50 | ***Sodium Citrate pH 6.0*** |
|  |  |  | ***1 in 100*** |  |
|  | Anti-CK | Dako | 1 in 150 | Sodium Citrate pH 6.0 |
| **Mouse/Rabbit** | Anti-CK/Anti-mouse biotin/HRP-Streptavidin | DAKO/DAKO/ Invitrogen | 1 in 50 /1 in 400 | Sodium Citrate pH 6.0 |
| **Mouse** | CK5/6/8/18 | Novocastra | 1 in 10 | TE Buffer pH 9.0 |
|  |  |  | 1 in 20 |  |
|  |  |  | 1 in 50 | Sodium Citrate pH 6.0 |
|  |  |  | 1 in 100 |  |
|  |  |  | 1 in 200 |  |
|  | CD10 | Novocastra | 1 in 10 | TE Buffer pH 9.0 |
|  |  |  | 1 in 20 |  |
|  |  |  | 1 in 50 | Sodium Citrate pH 6.0 |
|  |  |  | 1 in 100 |  |
|  |  |  | 1 in 200 |  |
|  | RCC | Novocastra | 1 in 10 | TE Buffer pH 9.0 |
|  |  |  | 1 in 20 |  |
|  |  |  | 1 in 50 | Sodium Citrate pH 6.0 |
|  |  |  | 1 in 100 |  |
|  |  |  | 1 in 200 |  |
|  | EMA | Dako | 1 in 10 | TE Buffer pH 9.0 |
|  |  |  | 1 in 20 |  |
|  |  |  | 1 in 50 | Sodium Citrate pH 6.0 |
|  |  |  | 1 in 100 |  |
|  |  |  | 1 in 200 |  |
|  | Anti-CK | Dako | 1 in 50 | Sodium Citrate pH 6.0 |
|  | Vimentin | Dako | 1 in 100 | Sodium Citrate pH 6.0 |
|  |  |  | 1 in 200 |  |
|  |  |  | 1 in 400 |  |
|  | Pan-cadherin | Sigma-Aldrich | 1 in 500 | Sodium Citrate pH 6.0 |
|  |  |  | 1 in 750 |  |
|  | Pan-cadherin | Sigma-Aldrich | 1 in 250 | TE Buffer pH 9.0 |
|  |  |  | 1 in 500 |  |
|  |  |  | 1 in 750 |  |
|  |  |  | 1 in 1000 |  |
|  | IL-15 | Abcam | 1 in 25 | Sodium Citrate pH 6.0 |
|  |  |  | 1 in 50 |  |
|  |  |  | 1 in 100 |  |
|  | Pan-cadherin/Anti-CK | Sigma-Aldrich/DAKO | 1 in 750 / 1 in 50 | TE Buffer pH 9.0 |
|  | ***Pan-cadherin/ CK5/6/8/18*** | ***Sigma-Aldrich/ Novocastra*** | ***1 in 750 / 1 in 100*** | ***TE Buffer pH 9.0*** |

Supplementary Table 3. The antibodies used during the tumour mask optimisation process and associated experimental conditions. Chosen, optimised protocols highlighted in bold.
