# Supplementary material for: The Use of Automated Quantitative Analysis to Evaluate Epithelial-to-Mesenchymal Transition Associated Proteins in Clear Cell Renal Cell Carcinoma
Source: PLoS One. 2012 Feb 21;7(2):e31557. doi: 10.1371/journal.pone.0031557 (PMC3283650; doi:10.1371/journal.pone.0031557)
Supplement: Table S4 — Comparison of patient pathology details and target Au scores with calculated uncorrected p-values (Kruskal–Wallis test, significance at p<0.05 level). Only grade 3 versus grade 4 remained significantly associated with E-Cadherin after correction for multiple comparisons (p = 0.004, significance at P<0.0083 level). (DOC) [file pone.0031557.s004.doc]

| **Variable** | **WT1 (nucleus)** | **phospho-β-catenin (nucleus)** | **phospho-β-catenin (cytoplasm)** | **SLUG (nucleus)** | **SNAIL (nucleus)** | **E-Cadherin (cytoplasm)** |
| --- | --- | --- | --- | --- | --- | --- |
| **Grade** | 0.203 | 0.175 | 0.353 | 0.458 | 0.192 | 0.032 |
| **Stage** | 0.636 | 0.326 | 0.288 | 0.263 | 0.747 | 0.888 |
| **Metastatic status** | 0.986 | 0.147 | 0.650 | 0.132 | 0.132 | 0.892 |
| **Nodal status** | 0.193 | 0.230 | 0.350 | 0.739 | 0.739 | 0.868 |

Supplementary Table 4. Comparison of patient pathology details and target Au scores with calculated uncorrected p-values (Kruskal–Wallis test, significance at p<0.05 level). Only grade 3 versus grade 4 remained significantly associated with E-Cadherin after correction for multiple comparisons (p=0.004, significance at P<0.0083 level).
