# Supplementary material for: The Use of Automated Quantitative Analysis to Evaluate Epithelial-to-Mesenchymal Transition Associated Proteins in Clear Cell Renal Cell Carcinoma
Source: PLoS One. 2012 Feb 21;7(2):e31557. doi: 10.1371/journal.pone.0031557 (PMC3283650; doi:10.1371/journal.pone.0031557)
Supplement: Table S5 — Area under the receiver operating characteristic curve (AUC) c-indexes for each clinicopathological variable and EMT biomarker. (DOC) [file pone.0031557.s005.doc]

| **Feature** | **Biomarker** | **Area under Curve (AUC)** | **95% Confidence Interval** |  |
| --- | --- | --- | --- | --- |
|  |  |  | *Upper Level* | *Lower Level* |
| **Node** | SLUG | 0.443 | 0.165 | 0.720 |
|  | E-Cadherin | 0.471 | 0.130 | 0.812 |
|  | SNAIL | 0.557 | 0.190 | 0.925 |
| **Grade** | SLUG | 0.440 | 0.295 | 0.585 |
|  | E-Cadherin | 0.537 | 0.390 | 0.684 |
|  | SNAIL | 0.581 | 0.433 | 0.729 |
| **Stage** | SLUG | 0.610 | 0.459 | 0.761 |
|  | E-Cadherin | 0.489 | 0.332 | 0.645 |
|  | SNAIL | 0.558 | 0.410 | 0.706 |
| **Metastasis status** | SLUG | 0.641 | 0.478 | 0.804 |
|  | E-Cadherin | 0.487 | 0.279 | 0.695 |
|  | SNAIL | 0.641 | 0.475 | 0.807 |
| **PFS status** | SLUG | 0.630 | 0.488 | 0.771 |
|  | E-Cadherin | 0.536 | 0.376 | 0.696 |
|  | SNAIL | 0.650 | 0.506 | 0.793 |

Supplementary Table 5. Area under the receiver operating characteristic curve (AUC) c-indexes for each clinicopathological variable and EMT biomarker.
